# Serum NGAL in Critically Ill Children in ICU from a Single Center in Egypt

**DOI:** 10.5402/2013/140905

**Published:** 2013-02-03

**Authors:** Doaa Mohammed Youssef, Asmaa Mohammed Esh, Ebthag Helmy Hassan, Tahia Mohammed Ahmed

**Affiliations:** ^1^Pediatric Department, Zagazig University, Zagazig 44519, Egypt; ^2^Clinical Pathology Department, Zagazig University, Zagazig 44519, Egypt

## Abstract

*Introduction.* The mortality and morbidity associated with acute kidney injury (AKI), unfortunately, remain unacceptably high. We aimed to detect the extent of serum neutrophil gelatinase-associated lipocalin (NGAL) to early detect AKI in critically ill children. *Subjects and Methods.* This is a case control study. It included 75 subjects that include 15 as controls and 60 critically ill children. Patients were further subdivided according to RIFLE criteria into two other categories: patients who developed AKI and patients who did not develop AKI. Serum NGAL assayed on admission and after 3 days. *Results.* There was significant increase in the level of NGAL among patients group when compared with control group. Also, 21.7% of children admitted to PICU developed AKI from which 8.3% needed dialysis. The receiver operating characteristic curve of NGAL at day 0 revealed AUC of 0.63 with 95% CI of 0.50–0.77. At a cutoff value of 89.5 ng/mL, the sensitivity of NGAL was 84.6%, while specifcity was 59.6%, positive predictive value was 36.7%, negative predictive value was 68.4%, and accuracy was 93.3% in diagnosis of AKI. *Conclusion.* We found that NGAL acts as a sensitive marker rather than a specific one for AKI. At the same time, it presents as a negative predictive value more valuable than being a positive predictive value in detecting AKI.

## 1. Introduction

Acute kidney injury (AKI), formerly known as acute renal failure, continues to represent a very common and potentially devastating problem in critically ill children and adults. The reported incidence of AKI in this population varies greatly due to the lack of a standard consensus definition. For example, AKI affects between 5% and 50% of critically ill patients in reported series [1].

Unfortunately, the mortality and morbidity associated with AKI remain unacceptably high (up to 80% mortality in critically ill children and adults with multiple organ dysfunction syndrome (MODS)). While this dismal prognosis is partly attributable to other comorbid conditions, recent studies have revealed that AKI may be an independent risk factor for mortality in both critically ill children [2] and adults [3].

Acute kidney injury is typically diagnosed by measuring serum creatinine. However, it is well known that creatinine is an unreliable and insensitive indicator during early acute changes in kidney function. First, serum creatinine concentrations may not change until about 50% of kidney function has already been lost. Second, serum creatinine does not accurately depict kidney function until a steady state has been reached, which may require several days [4].

Neutrophil gelatinase-associated lipocalin is a 25 kDa protein that is expressed at very low concentrations in several human tissues, including the kidney, lungs, and gastrointestinal tract [5].

The aim of this study was to detect the extent of serum neutrophil gelatinase-associated lipocalin (NGAL) to early detect acute kidney injury (AKI) in critically ill children and to evaluate its sensitivity and specificity in AKI detection.

## 2. Subjects and Methods

This study had been carried out in Pediatric Intensive Care Unit (PICU) and Clinical Pathology Department, Faculty of Medicine, Zagazig University Hospital. 

This study included (75) subjects. They were classified into group I (15), apparently healthy volunteers, and their ages ranged between 1 and 29 months with the median age of 6 months, and group II (60), children who were admitted at PICU, and their ages ranged between 1 and 108 months with the median age of 9 months.

For group I routine investigations as liver function, kidney function, and complete blood count were done to confirm their healthy state.

 For group II, serum creatinine was measured in the first day of admission, and then another sample was measured in the 3rd day of admission to the PICU. This group of patients was further subdivided according to RIFLE criteria into two other categories: group A, patients who developed AKI (AKI) and group B, patients who did not develop AKI (NAKI). The RIFLE criteria include (risk of acute renal failure, injury to the kidney, and failure of renal function).

Then, the patients who developed AKI are further divided into two other groups according to the severity (dialysis dependence) as follow: AKI with dialysis (AKI + D) and AKI without dialysis (AKI − D). 

All studied subjects included in this study were subjected to the following: full history taking, including family history of underlying kidney disease, complete clinical examination, routine laboratory investigations, and serum NGAL assay.

### 2.1. NGAL Assay

#### 2.1.1. Sampling

Five milliliters of venous blood were collected under complete aseptic precautions from each subject. The collected blood was divided as the following.

- 1 mL was delivered into EDTA containing polypropylene tube and then gently inverted, then CBC was performed.
- 4 mL were collected in plain tube for serum separation. After clotting, samples were centrifuged at 1000 ×g for 15 minutes, and sera were separated and divided into two aliquots. A fresh serum aliquot from each individual was used for assay of serum creatinine, blood urea nitrogen (BUN), and liver function tests. The other aliquot was stored at −20°C until the assay of neutrophil gelatinase-associated lipocalin. Hemolyzed samples were discarded, and repeated freezing and thawing was avoided.

### 2.2. NGAL Assay by Enzyme-Linked Immunosorbent Assay

Assay of NGAL was carried out by a sandwich enzyme-linked immunosorbent assay (ELISA) technique using reagents provided by Quantikine R&D International Inc. (R&D Systems, Inc., MN, USA). 

*Principle.* This assay employs the quantitative sandwich enzyme immunoassay technique. A monoclonal antibody specific for NGAL has been precoated onto a microplate. Standards and samples are pipetted into the wells, and any NGAL present is bound by the immobilized antibody. After washing away any unbound substances, an enzyme-linked monoclonal antibody specific for NGAL is added to the wells. Following a wash step to remove any unbound antibody enzyme reagent, a substrate solution is added to the wells, and color develops in proportion to the amount of NGAL bound in the initial step. The color development is stopped, and the intensity of the color is measured [6].

*Reagents are as follows*

- NGAL microplate: 96-well polystyrene microplate (12 strips of 8 wells) coated with a rat monoclonal antibody against NGAL;
- NGAL conjugate: 21 mL of monoclonal antibody against NGAL conjugated to horseradish peroxidase with preservatives;
- NGAL standard: 100 ng of recombinant human NGAL in a buffer with preservatives lyophilized. NGAL standard was reconstituted with 1.0 mL of deionized or distilled water. This reconstitution produces a stock solution of 100 ng/mL. The standard was mixed to ensure complete reconstitution, and the standard was allowed to sit for a minimum of 15 minutes with gentle agitation prior to making dilutions.

Serial dilution of standard was done to obtain the following concentrations: 10, 5, 2.5, 1.25, 0.625, 0.312, and 0.156 ng/mL. 900 *μ*L of calibrator diluent were pipetted into the 10 ng/mL tube. 500 *μ*L were pipetted into the remaining tubes. The stock solution was used to produce a dilution series (later). Each tube was mixed thoroughly before the next transfer. The 10 ng/mL standard served as the high standard. The calibrator diluent served as the zero standard (0 ng/mL);

(iv)  assay diluent RD1-52: 11 mL of a buffer with preservatives;
(v)  calibrator diluent RD5-24 concentrate: 21 mL of a 5-fold concentrated solution containing buffered protein base with preservatives;
(vi)  wash buffer concentrate: 21 mL of a 25-fold concentrated solution of buffered surfactant with preservatives;
(vii)  color reagent A: 12.5 mL of stabilized hydrogen peroxide;
(viii)  color reagent B: 12.5 mL of stabilized chromogen (tetramethylbenzidine);
(ix)  stop solution: 6 mL of 2 N sulfuric acid;
(x)  plate covers: 4 adhesive strips.

*Assay Procedure*

- 100 *μ*L of assay diluent RD1-52 were dispensed into each well.
- 50 *μ*L of standards, controls, and samples were added to each well.
- The plate was covered and incubated for 2 hours at 2–8°C.
- Aspiration of each well was done and this was followed by a washing step which was repeated three times.
- 200 *μ*L of NGAL conjugate were added into each well, covered with a new adhesive strip and incubated for 2 hours at 2–8°C.
- Aspiration/washing was repeated as in step (iii).
- 200 *μ*L of substrate solution were dispended into each well.
- Plate was covered and incubated for 30 minutes at room temperature.
- 50 *μ*L of stop solution were added into each well.
- Determination of the optical density of each well within 30 minutes was done, using a microplate reader set to 450 nm.

*Calculation of Results.* A standard curve was constructed by plotting the mean absorbance for each standard on the *y*-axis against the concentration on the *x*-axis on log-log graph, and a best fit line was drawn through the points on the graph. Concentration of lipocaline-2 in each sample was determined by finding the mean absorbance value of each one. From the *y*-axis of the standard curve graph, a vertical line was extended from this absorbance value to the standard curve. At the point of intersection, a horizontal line was extended to the *x*-axis and the corresponding concentration was read.

## 3. Statistical Analysis

The data were tabulated and statistically analyzed using Statistical Package for Social Sciences (SPSS Inc., Chicago, IL, USA) version 17. Comparison of continuous data was performed using student *t*-test and Mann-Whitney test for two groups, while for more than two groups, ANOVA test was used. ROC curves analysis (an efficient way to display the relationship between sensitivity (true positive rate) and specificity (true negative rate) for tests with continuous outcomes; a point in the curve moves down and to the left showing lower sensitivity and high specificity, while it moves up and to the right showing higher sensitivity and lower specificity) was used. The cutoff value was selected from the ROC curve by choosing a point having the best sensitivity and considerable specificity. *P* value < 0.05 was considered statistically significant.* Sensitivity* is the proportion of true positive that are correctly identified. *Specificity *is the proportion of true negative that are correctly identified. *Positive predictive Value (PPV)*  is  probability of disease in a patient with an abnormal test. *Negative Predictive Value (NPV)*  is  probability of a patient not having the disease when the test result is negative.

## 4. Results

The routine Laboratory results in our studies subjects were presented on Table 1. We found that range of NGAL at day zero was (34–210) with median of 88.5 and at 3rd day was (34–550) with median 114, urea at day zero ranged from 15 to 116 with median of 35.5 and at 3rd day ranged from 27 to 310 with median of 41.5, while creatinine at zero day was (X′ ± SD: = 0.68 ± 0.22) while at 3rd day (X′ ± SD: 1.18 ± 1.08). So, there were statistical significance between NGAL, urea, and creatinine at day zero and 3rd day (*P* = 0.000) as shown in Table 2.

A significant increase between two groups with and without AKI regarding NGAL, urea, and creatinine at admission and at 3rd day (*P* < 0.05) was shown in Table 3.

There was no statistical significance among the 3 groups regarding NGAL at admission and urea at 3rd day (*P* = 0.24 and 0.76, resp.). However, there was statistical significance among the 3 groups regarding NGAL at 3rd day, urea at admission and creatinine at admission, and at 3rd day (*P* = 0.07, 0.01, 0.004, and 0.000 resp.) as presented in Table 4.

**Figure 1:**
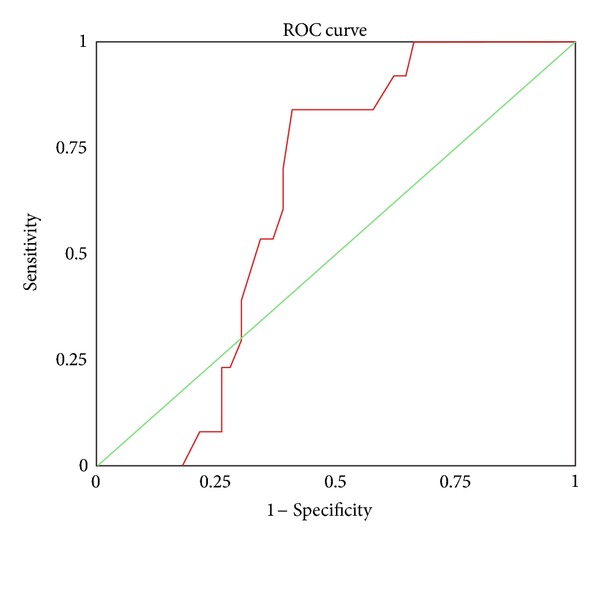
Receiver operating characteristic curve of NGAL.

Table 5 described that applying the receiver operating characteristic (ROC) curve of NGAL to early detection of AKI revealed area under the curve of 0.63 with 95% confidence interval (CI) of 0.50–0.77. A cutoff of 89.5 ng/mL was chosen for early detection of AKI presented in Figure 1.

NGAL acts as a sensitive marker rather than a specific one. At the same time, it presents a negative predictive value more valuable than being a positive predictive value in detecting AKI. We described in Table 6 that, at cutoff of 89.5 ng/mL, NGAL was positive at 11 out of 13 patients with AKI; the sensitivity of NGAL in diagnosis of AKI was 84.6%. Howerver, 28 out of 47 had truly absent AKI with specificity 59.6%. The positive predictive value (11/(11 + 19)) was 36.7%, and the negative predictive value (28/(2 + 28)) was 93.3%. 

## 5. Discussion

Acute kidney injury is a common and serious condition, and the diagnosis of which depends on serum creatinine urea which is a delayed and unreliable indicator of AKI. Fortunately, understanding the early stress response of the kidney to acute injuries has revealed a number of potential biomarkers [7].

Neutrophil gelatinase-associated lipocalin is a small protein of the lipocalin superfamily and is expressed by immune cells, hepatocytes, and renal tubular cells [8]. 

In particular, NGAL is emerging as an excellent biomarker in the urine and plasma for several processes such as early prediction of AKI, monitoring clinical trials in AKI, and for the prognosis of AKI in several common clinical scenarios [7].

The aim of the present work was to assess serum levels of NGAL in critically ill children affected by AKI and to evaluate its clinical significance in diagnosis as well as assessment of disease severity in comparison with other markers as serum creatinine and urea ratio in critically ill children.

We prospectively measured serum NGAL concentrations during day zero and after the 3rd day of admission to the PICU in 60 critically ill children (28 males and 32 females) and 15 healthy children (8 males and 7 females). 

In our prospective study, there were no statistical significant differences between controls and patients group regarding demographic data including age and gender, *P* = 0.15, and *P* = 0.64, respectively. 

The liver function (total bilirubin, direct bilirubin, and ALT) showed that there was no statistical significance difference between critically ill children and control group. However, there was a significant increase in the level of AST and significant decrease in the levels of albumin and total protein among the two groups. 

Regarding kidney function, there was significant increase in the level of urea in patients group compared to control group (*P* = 0.000). However, creatinine value increased in critically ill children group showing no statistical significance with control group (*P* = 0.69).

This is in agreement with Bagshaw et al. (2006) [3] who reported that urea is not produced at a constant rate, and the rate can be influenced by extrarenal factors. Urea production can be increased by diet, critical illness, burns, trauma, gastro intestinal bleeding, and sepsis. Also, in patients with decreased circulating blood volume due to volume depletion or low cardiac output, resorption of urea increases because of the relationship between urea levels and water conservation mechanisms. Therefore, urea can be influenced by multiple factors and does not represent real-time changes in GFR. 

In the present study, the level of serum NGAL concentration for control group ranged from 44 to 141 ng/mL with median of 54 ng/mL and critically ill children group ranged from 34 to 210 ng/mL with median of 88.5 ng/mL at admission. There was significant increase in the level of NGAL in the critically ill children group compared to control group, *P* < 0.01.

These findings are in agreement with Bailey et al. (2007) who reported that there was a significant difference in serum NGAL between healthy children and critically ill children [1].

Critically ill children who developed AKI (by doubling of serum creatinine from baseline, according to RIFLE classification) had significantly elevated serum NGAL, urea, and creatinine levels at 3rd-day compared to serum NGAL, urea, and creatinine at day zero. Also, critically ill children who developed AKI had significantly elevated serum NGAL, urea, and creatinine levels at day zero and at 3rd day when compared with critically ill children who did not develop AKI.

This goes hand in hand with Mishra et al. (2005) [8] and Wheeler et al. (2008) [9] who demonstrated that serum NGAL concentrations within 24 hours of PICU admission were significantly increased in these children who developed AKI compared to children who did not develop AKI. A second sample was obtained on the third day of admission to the PICU, and the difference between critically ill children with AKI and critically ill children without AKI remained significant (*P* < 0.001).

AKI developed in 13 out of 60 (21.7%) critically ill children included in this study—all but 5 of these critically ill children (8.3%) had a greater severity of illness and need to dialysis. Patients who developed AKI and need to dialysis showed higher level of NGAL at day 0 compared to patients who developed AKI without need to dialysis and patients who did not develop AKI, but with no statistical significance, while at 3rd day, it markedly increased (median = 350 ng/mL) and became highly significant (*P* = 0.04). 

These findings are in agreement with Hoste et al. (2003) [10] who found that AKI developed in 32 out of 69 (46.3%) critically ill children—all but 8 of these critically ill children (11.5%) had a greater severity of illness and need to dialysis.

In our study, the receiver operating characteristic curve of NGAL for early detection of AKI revealed area under the curve of 0.63 with 95% (CI of 0.50–0.77). At cutoff value of 89.5 ng/mL, the sensitivity of NGAL was 84.6%, the specificity was 59.6%, positive predictive value was 36.7%, and negative predictive value was 68.4%.

Close results were found by Akcan-Arikan et al. (2007) [2] who had shown that at cutoff of 139 ng/mL, the receiver operating characteristic curve of NGAL revealed area under the curve of 0.677 with 95% (CI of 0.557–0.786). The sensitivity of NGAL was 86%, specificity was 39%, positive predictive value was 39%, and negative predictive value was 94%.

Our results of early predictive, sensitive, nonspecific serum NGAL, although of clear statistical significance, will certainly need to be validated in a larger trial, including patients with preexisting chronic kidney disease and co morbid conditions that normally accumulate with impaired renal function. The ability of biomarkers, such as NGAL, to discern both the onset and resolution of AKI will further validate their use in the clinical setting and greatly enhance our understanding of AKI in the pediatric population. 

## 6. Conclusion

In conclusion, we found that NGAL acts as a sensitive marker rather than a specific for one AKI. At the same time, it presents a negative predictive value more valuable than being a positive predictive value in detecting AKI.

## Figures and Tables

**Table 1:** Laboratory investigations of study groups regarding liver and kidney functions.

Parameter	Control *N* = 15	Patients *N* = 60	Test of significance	*P*
Total bilirubin (mg/dL)				
Median	0.4	0.5	1.25^b^	0.210
Range	(0.3–0.8)	(0.3–23.4)		
Direct bilirubin (mg/dL)				
Median	0.1	0.5	0.99^b^	0.321
Range	(0.1–0.2)	(0.1–12.9)		
ALT (U/L)				
Median	31	45.5	1.11^b^	0.26
Range	(12–42)	(9–133)		
AST (U/L)				
Median	38	55.6	3.007^b^	0.003*
Range	(5–55)	(18–1207)		
Albumin (g/dL)				
X′ ± SD	(3.907 ± 0.371)	(3.36 ± 0.618)	3.245^a^	0.002*
TP (g/dL)				
X′ ± SD	(6.8 ± 0.442)	(5.7 ± 0.868)	4.47^a^	0.001*
Urea (mg/dL)				
Median	25.8	35.5	3.42^b^	0.001*
Range	(15–37)	(15–116)		
Creatinine (mg/dL)				
X′ ± SD	0.65 ± 0.22	0.68 ± 0.22	0.38^a^	0.69
WBCs (×103/cmm)	12.1 (6.1–14.1)	13.1 (2.1–56.2)	0.64^b^	0.51
Hb (g/dL )	12.5 (9.5–15.5)	10.5 (6.6–18)	6.26^b^	0.001*
Platelet (×109/cmm)	250 (180–340)	222 (35–1008)	0.49^b^	0.62
NGAL-0 (ng/mL)	54 (44–141)	88.5 (34–210)	2.47^b^	0.01*

Test of significance: ^a^ *t*-test; ^b^Mann-Whiney test; *significant.

**Table 2:** Acute kidney injury (AKI) markers at admission (day 0) and after 3 days (3rd day) among patients group.

Parameter	Day zero	3rd day	Wilcoxon's rank sum test	*P*
NGAL (ng/mL)				
Median	88.5	144	6.28	0.000*
Range	(34–210)	(34–550)		
Urea (mg/dL)				
Median	35.5	41.5	6.01	0.000*
Range	(15–116)	(27–310)		
Creatinine				
(mg/dL)				
X′ ± SD	0.68 ± 0.22	1.18 ± 1.08	4.1	0.000*

*Significant.

**Table 3:** Serum NGAL, urea, and creatinine at day zero and 3rd day among 2 groups of patients: AKI (group A) and NAKI (group B).

Parameters	AKI		Test of significance	*P*
	Absent *N* = 47	Present *N* = 13		
NGAL-0 (ng/mL)				
Median	76	110	2.64^b^	0.008*
Range	(34–200)	(66–210)		
NGAL-3 (ng/mL)				
Median	105	280	3.32^b^	0.001*
Range	(34–530)	(92–550)		
Urea-0 (mg/dL)				
Median	34.5	42.5	2.04^b^	0.040*
Range	(15–116)	(28.7–78.8)		
Urea-3 (mg/dL)				
Median	38	113.4	3.00^b^	0.003*
Range	(18–310)	(7–228)		
Creatinine-0 (mg/dL)				
X ± SD	0.61 ± 0.23	0.86 ± 0.23	3.4^a^	0.001*
Creatinine-3 (mg/dL)				
X ± SD	0.73 ± 0.63	2.08 ± 1.24	10.1^a^	0.000*

Test of significance: ^a^ *t*-test; ^b^Mann-Whiney test; *significant.

**Table 4:** Comparative study of patients group according to NGAL, Urea, and creatinine at admission (day zero) and 3rd day and their classification according to severity to 3 subgroups: NAKI, AKI − D, and AKI + D.

Parameter	NAKI	AKI − D	AKI + D	Test of significance	*P*
	*N* = 47	*N* = 8	*N* = 5		
NGAL-0 (ng/mL)					
Median	84	100.5	120	1.37^b^	0.24
Range	(34–210)	(74–115)	(66–148)		
NGAL-3 (ng/mL)					
Median	105	184	350	3.08^b^	0.04*
Range	(34–530)	(42–395)	(150–550)		
Urea-0 (mg/dL)					
Median	34.5	38	65	5.91^b^	0.01*
Range	(15–116)	(28–57)	(42.5–788)		
Urea-3 (mg/dL)					
Median	38	84.1	113.4	0.08^b^	0.76
Range	(18–310)	(40–228)	(7–178)		
Creatinine-0 (mg/dL)					
X ± SD	0.6 ± 0.23	0.8 ± 0.27	0.94 ± 0.15	*F* = 6.22	0.004*
Creatinine-3 (mg/dL)					
X ± SD	0.73 ± 0.36	2.25 ± 1.01	3.7 ± 1.1	*F* = 77.9	0.000*

^b^Mann-Whitney test; *F*: Fisher test; *significant.

**Table 5:** Area under the receiver operating characteristic curve (ROC-AUC) and cutoff value for NGAL.

Parameter	ROC-AUC	95% CI	Cutoff
NGAL-0	0.63	0.50–0.77	89.5 ng/mL

**Table 6:** Validity of NGAL in prediction of AKI.

Parameter	AKI		Total
	Present	Absent
NGAL ≥ 89.5	11	19	30
NGAL ≤ 89.5	2	28	30

Total	13	47	60

## References

[1] Bailey D, Phan V, Litalien C (2007). Risk factors of acute renal failure in critically ill children: a prospective descriptive epidemiological study. *Pediatric Critical Care Medicine*.

[2] Akcan-Arikan A, Zappitelli M, Loftis LL, Washburn KK, Jefferson LS, Goldstein SL (2007). Modified RIFLE criteria in critically ill children with acute kidney injury. *Kidney International*.

[3] Bagshaw SM, Langenberg C, Bellomo R (2006). Urinary biochemistry and microscopy in septic acute renal failure: a systematic review. *American Journal of Kidney Diseases*.

[4] Moran SM, Myers BD (1985). Course of acute renal failure studied by a model of creatinine kinetics. *Kidney International*.

[5] Kjeldsen L, Johnsen AH, Sengelov H, Borregaard N (1993). Isolation and primary structure of NGAL, a novel protein associated with human neutrophil gelatinase. *The Journal of Biological Chemistry*.

[6] Mori K, Nakao K (2007). Neutrophil gelatinase-associated lipocalin as the real-time indicator of active kidney damage. *Kidney International*.

[7] Devarajan P (2011). Biomarkers for the early detection of acute kidney injury. *Current Opinion in Pediatrics*.

[8] Mishra J, Dent C, Tarabishi R (2005). Neutrophil gelatinase-associated lipocalin (NGAL) as a biomarker for acute renal injury after cardiac surgery. *The Lancet*.

[9] Wheeler DS, Devarajan P, Ma Q (2008). Serum neutrophil gelatinase-associated lipocalin (NGAL) as a marker of acute kidney injury in critically ill children with septic shock. *Critical Care Medicine*.

[10] Hoste EAJ, Lameire NH, Vanholder RC, Benoit DD, Decruyenaere JMA, Colardyn FA (2003). Acute renal failure in patients with sepsis in a surgical ICU: predictive factors, incidence, comorbidity, and outcome. *Journal of the American Society of Nephrology*.

